# Host inflammatory response and clinical parameters around implants in a rat model using systemic alendronate and zoledronate acid drug administrations

**DOI:** 10.1038/s41598-022-08308-8

**Published:** 2022-03-15

**Authors:** Kristian Kniha, Lothar Rink, Jana Wolf, Stephan Christian Möhlhenrich, Florian Peters, Marius Heitzer, Frank Hölzle, Ali Modabber

**Affiliations:** 1 Department of Oral and Cranio-Maxillofacial Surgery, University Hospital RWTH Aachen, University Aachen, Pauwelsstraße 30, 52074 Aachen, Germany; 2 Institute of Immunology, University Hospital RWTH Aachen, Pauwelsstraße 30, Aachen, Germany; 3 Department of Orthodontics, University of Witten/Herdecke, Alfred-Herrhausen Str. 45, 58455 Witten, Germany

**Keywords:** Immunology, Medical research

## Abstract

Implant outcomes in comparison to a natural tooth in a rat model using systemic alendronate and zoledronate acid drug administrations were assessed. Fifty-four Sprague–Dawley rats were randomly allocated into two experimental groups (drug application of zoledronic acid; 0.04 mg/kg intravenously once a week and alendronic acid; 0.2 mg/kg subcutaneously five times a week) and one control group with 18 animals in each group. Drug delivery was conducted for a period of 4 months. After 4 weeks either a zirconia or a titanium implant was immediately inserted in the socket of the first molar of the upper jaw. In vivo investigations included host inflammatory parameters and the implant survival and success rates for up to 3 months. Material incompatibilities against titanium and zirconia nanoparticles were evaluated in vitro after stimulation of rat spleen cells. In vivo, IL-6 release around titanium implants demonstrated significantly higher values in the control group (p = 0.02) when compared to the zoledronic acid group. Around the natural tooth without drug administration, the control group showed higher IL-6 values compared with the alendronic acid group (p = 0.01). In vitro, only lipopolysaccharide and not the implant’s nanoparticles stimulated significant IL-6 and TNFα production. In terms of the primary aim of in vivo and in vitro IL-6 and TNFα measurements, no implant material was superior to the other. No significant in vitro stimulation of rat spleen cells was detected with respect to titanium oxide and zirconium oxide nanoparticles.

## Introduction

In clinical dental practice, dentists increasingly encounter patients who regularly take bisphosphonates^1^. Osteoblasts and osteoclasts communicate with each other via different signaling molecules^2^. The osteoblasts produce a protein called RANKL, which after binding to the RANK receptor on the osteoclast activates the osteoclasts so that bone is resorbed^2^. Bisphosphonates are pyrophosphate analogues in which oxygen is substituted by carbon in the P-O-P bond^3^. As a result, no enzymatic hydrolysis occurs in the body. Bisphosphonates have a very high affinity for bone mineral because they bind to hydroxyapatite crystals^4^. Medication-related osteonecrosis of the jaw (MRONJ) has been described as a side effect^5^. Different pathologies have been discussed in the development of MRONJ^6^. On the one hand, reduced bone remodeling was described as the main cause. The inhibition of farnesyl-diphosphatase inhibits osteoclasts and leads to an increased rate of apoptosis of the osteoclasts^2^. Bisphosphonates also promote the reduction of vascular cells and thus the development of avascular necrosis, since the vascular supply is essential for a vital bone metabolism^7,8^. The medicinal effect influences not only the bone but also the overlying soft tissue, which explains the accompanying wound-healing disorders in these patients^7,9^. Systemic dosages are also perceived to have effects on the osteoblasts, which are also inhibited^7^. Not only bisphosphonates, but also other drugs, such as monoclonal antibodies, vascular endothelial growth factors, and tyrosine kinase inhibitors, have been related to MRONJ^6,10,11^. The prevalence of MRONJ, depending on the underlying disease and the type of drug administration, could be as high as 20% in intravenous injections in the case of malignant underlying disease. By contrast, oral drug administrations lead to low risk profiles; for example, primary osteoporosis at approximately 0.1%^7^. Bisphosphonates have a very long pharmacological half-life in the bone: in some cases more than ten years, which means that the indication for the use of this active substance should be strict^5^. Implant placement can be a risk factor. There are numerous case series of osteonecrosis associated with bisphosphonate after implantation or retrospective studies of bisphosphonate-associated osteonecrosis that have observed a relationship between osteonecrosis and bisphosphonate^12–16^. On the other hand, a number of studies have not observed any such connection^17–21^. The alternative therapy to implantological solutions often represents a purely tegumentally supported dental prosthesis, but here too, studies show a clear relationship between prostheses and their pressure points to the occurrence of osteonecrosis^22–24^. These studies only included titanium implants. Whether modern zirconium dioxide implants have advantages due to other material properties, such as a reduced tendency to mucositis, remains unclear^25,26^. It is of great importance to learn more about modern zirconia implants, especially with regard to the material properties and the resulting individual peri-implant inflammation risk. Proinflammatory cytokines such as TNF alpha, that promotes bone resorption and mediates the inflammatory response to infection, can be used to evaluate the level of inflammation^27^. The connection between soft tissue inflammation and the expression of inflammation-associated biomarkers around the teeth and implants has been demonstrated previously^28,29^. The purpose of this rat study was to investigate the peri-implant interface around titanium and zirconia implants in a high-risk group with systematic antiresorptive bisphosphonate drug administration. The primary aim was to assess immunological parameters, such as interleukin-6 (IL-6) and tumor necrosis factor alpha (TNFα), after immediate implant insertion in comparison to the natural tooth. The secondary aim was to conduct an in vitro stimulation test to evaluate hypersensitivity reactions to titanium and zirconium oxide nanoparticles. Furthermore, implant success rates and clinical parameters were evaluated.

## Material and methods

### Experimental protocol

The local authority office has approved the study. Fifty-four adult male Sprague–Dawley rats with a weight of 250 g and aged 7 weeks (Janvier Labs, Le Genest-Saint-Isle, France) were included in this study. This investigation was conducted in accordance with the guidelines of the European Parliament and of the Council on the protection of animals used for scientific purposes, Directive 2010/63 EU and was related to the immunological and clinical findings. We confirm that all experiments were performed in accordance with relevant named guidelines and regulations and the study complied with the ARRIVE guidelines. The study protocol was approved by the appropriate local authority (Landesamt für Natur und Verbraucherschutz, Recklinghausen, Germany; Ref. 2018A314). According to the laws in Germany § 15 TierSchG, the local ethic committee of the RWTH university does not provide permission to animal studies in Nord-Rhein Westfalen, Germany, only the LANUV https://www.lanuv.nrw.de/verbraucherschutz/tierversuche.

**Figure 1:**
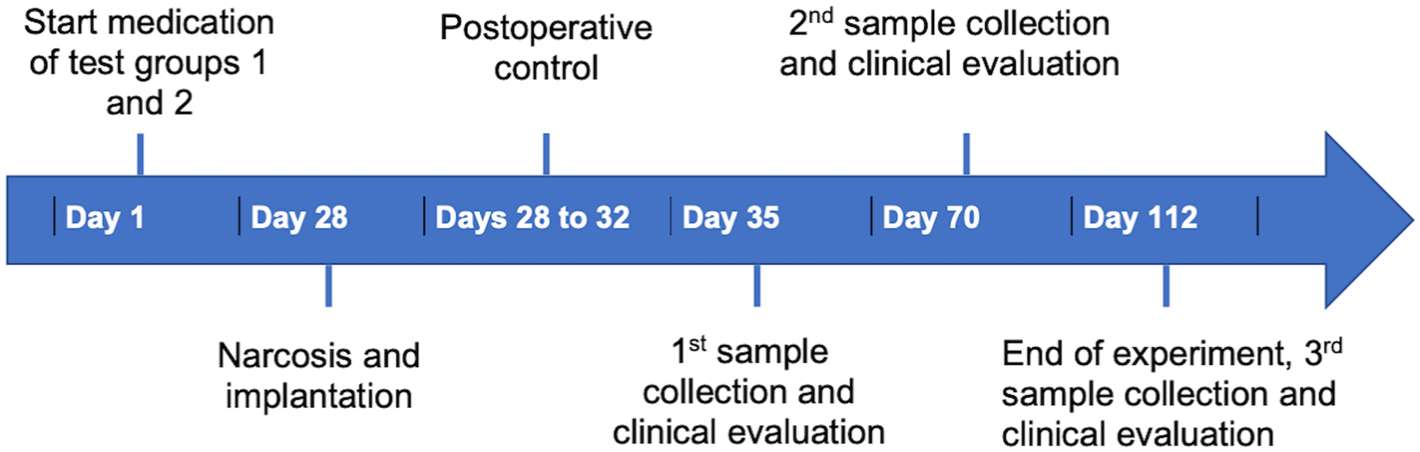
Timeline of the experiment.

A single examiner performed each assessment for clinical and immunological diagnosis during the entire period of the study. Two experimental groups and one control group with 18 animals in each group were randomly divided as follows: one group treated with zoledronic acid, one group treated with alendronic acid, and one control group without any drug administration. Systemic drug administration with antiresorptive drugs was conducted for a period of 4 months and was started 4 weeks before surgery (Fig. 1). The drugs were diluted with physiologic phosphate-buffered saline before application. The zoledronic acid group received a dose of 0.04 mg/kg of their body weight of zoledronic acid (Mylan dura GmbH, Darmstadt, Germany) intravenously in the tail vein once every week^30^. In the second test group, a total of 0.2 mg/kg body weight of alendronic acid (Alendronate sodium trihydrate, Sigma Aldrich GmbH, Munich, Germany) was applied subcutaneously five times a week^31^. The rats were provided with food and water ad libitum, with only soft soaked food administered after implantation until the end of the investigation.

### Implant placement

A total of 54 microrough titanium and 54 zirconia implants with a polished shoulder (length of 4 mm and diameter of 2 mm) were custom-made by Straumann Company with the same process used on commercially available implants (Institute Straumann AG, Basel, Switzerland).

**Figure 2:**
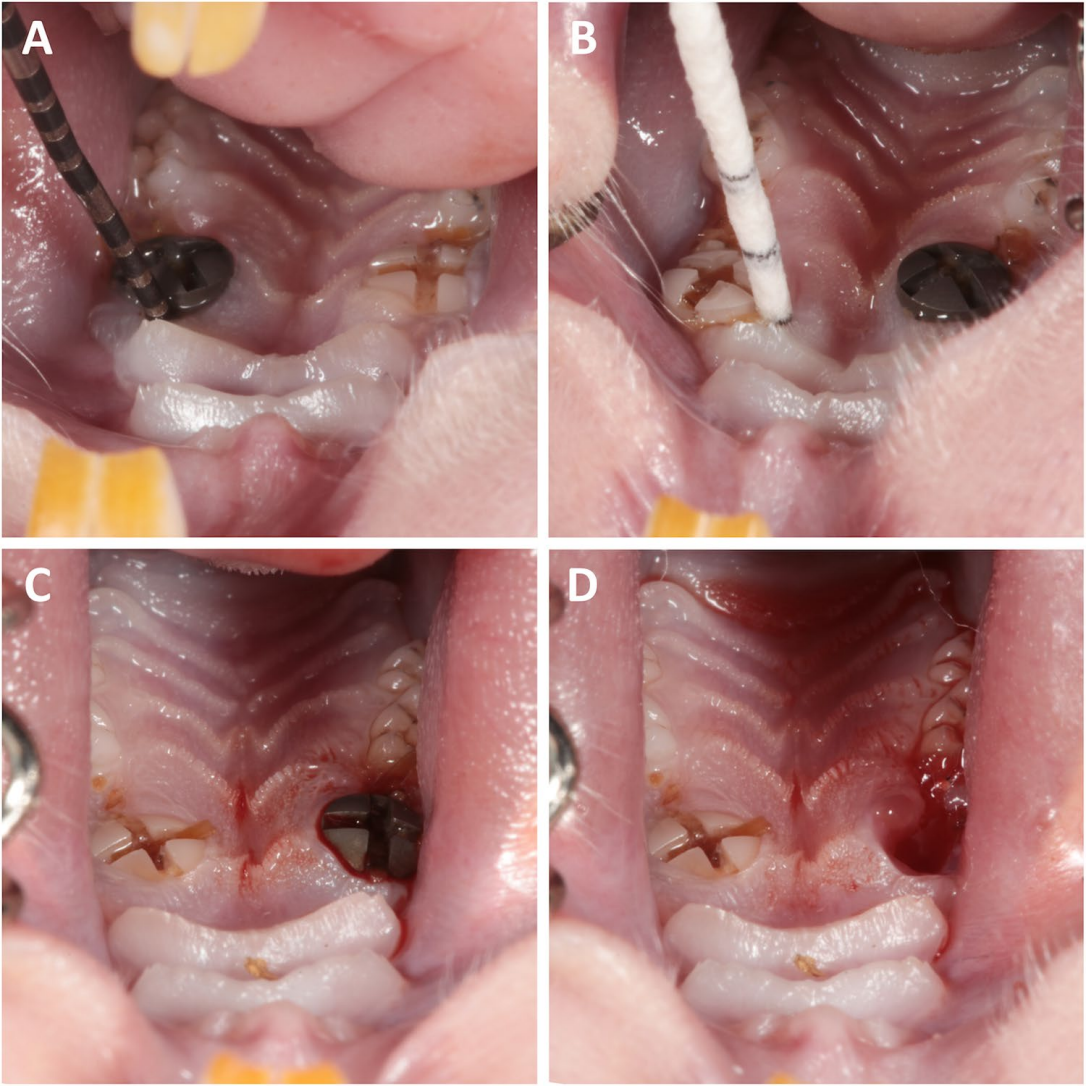
(**A**) Intraoral view of the upper jaw. The clinical probing pocket depth was measured with a dental probe. (**B**) In vivo material were collected with sterile color-coded paper points. (**C**) The sulcus bleeding index was evaluated around each implant (SBI around the titanium implant was 3 = severe bleeding). (**D**) In this case due to peri-implantitis and loss of stability, the titanium implant had to be removed.

After 4 weeks of drug delivery, the rats received an intraperitoneal anesthetic cocktail consisting of 90 mg/kg of body weight of ketamine (Medistar GmbH, Ascheberg, Germany) and 0.2 mg/kg of body weight of medetomidine hydrochloride (Domitor, Bayer Austria, Wien, Austria). Subsequently, after extraction of the first molar of the upper jaw on each side, either a zirconia or a titanium implant was immediately inserted using a randomization of the side distribution (Fig. 2). The insertion process included a pilot drill with a 2.2-mm diameter (Institute Straumann AG, Basel, Switzerland) and was performed according to the manufacturer’s protocol using a screwdriver, a torque of 15–20 Ncm, and a transgingival healing process. At the end of the surgery, the antidote atipamezole hydrochloride (Orion Pharma, Espoo, Finland), at a dose of 0.8 mg/kg body weight, was applied subcutaneously. In the first postoperative 3 days, the animals were visited and treated once a day with carprofen (4 mg/kg) subcutaneously (Rimadyl, Zoetis GmbH, Berlin, Germany) according to a score sheet.

### In vivo immunological and clinical examination

In vivo sample collection and clinical inspection of the rats were conducted under inhalation anesthesia with isofluran (2.5–5 vol.% Piramal GmbH, Hallbergmoos, Germany) at three different time points (1 week, 8 weeks, and 12 weeks after surgery, Fig. 1).

Before the sample collection, under sterile conditions, the paper points with color-coded ends were cut off at the lowest colored end, according to the procedure of a previously published study^25^. Afterwards, sterile paper points were inserted at the deepest probing pocket depth around each implant (either zirconia or titanium) or natural tooth (second molar of one side), which was the deepest side at baseline measurements (VDW, 29 mm, ISO 15, Taper.04, Munich, Germany). The defined tip between two marks was cut after the gingival/peri-implant mucosal crevicular fluid was sampled up to the second colored end. Afterwards, the samples were stored in tubes filled with 350 µl Dulbecco’s phosphate buffer saline without CaCl_2_ and MgCl_2_ (PBS, Sigma-Aldrich, Steinheim, Germany) + 10% fetal calf serum (FCS, Lonza, Verviers, Belgium) at − 80 °C. After sample assessment, a calibration curve for recovery ELISA standard with paper points was applied. Additionally, the lowest standard parameters for each value were assessed and projected to the dilution of 1:351 with PBS + 10% FCS. All samples were thawed only once for analysis. Cytokines were detected by ELISA as per manufacturer’s instructions. BD Rat ELISA sets were used to measure IL-6 and TNFα (BD Biosciences Pharmingen, San Diego, CA, USA). ELISA was quantified on TECAN Spark 10 M (TECAN AG, Männedorf, Switzerland). The final cytokine concentrations were calculated by multiplication with the dilution factor (× 351) in the storage buffer and the defined recovery coefficient (× 8.52 for IL-6 and × 1.79 for TNF). Detection limits were defined in the same manner using the lowest positive standard of the respective ELISA (0.078 ng/ml for IL-6 and 0.031 ng/ml for TNF).

Clinical parameters included the pocket probing depth (PPD) in mm from the bottom of the sulcus to the gingival/mucosal margin using a Michigan periodontal probe at four points around each unit (Fig. 2A, at sessions 1, 2, and 3, 1 week, 8 weeks, and 12 weeks after surgery)^32–34^. Additionally, the sulcus bleeding index (SBI, Saxer & Mühlemann, score 0 = no bleeding, 1 = isolated bleeding, 2 = confluent linear bleeding, and 3 = severe bleeding) was investigated (sessions 1, 2, and 3). The survival rate showed whether the implant was still present in the animal’s mouth at the time of examination. Implant success was based on the criteria of Jahn and d'Hoedt^35^. The various success parameters were no implant mobility (in case of any implant mobility, it was considered not successful), no clinical infection (such as gingival inflammation, swelling, secretion and redness of the tissue) or bleeding on probing less than score 2 (0 = no bleeding, 1 = isolated bleeding, 2 = confluent linear bleeding, and 3 = severe bleeding), or increased PPD (the sulcus depths must not exceed 2 mm for 2 consecutive controls)..

### In vitro immunological examination

IL-6 and TNFα levels were evaluated after stimulation of rat spleen cells with different materials. After finalization of the animals, 18 rat spleens of the control group were directly prepared and stored in RPMI 1640 without L-Glutamine (Sigma-Aldrich, Steinheim, Germany) with 10% FCS (see above), 2 mM L-Glutamine (Sigma-Aldrich, Steinheim, Germany), 100 U/ml Penicillin (Sigma-Aldrich, Steinheim, Germany) and 100 µg/ml Streptomycin (Sigma-Aldrich, Steinheim, Germany). The rat spleen of one rat without any cellular contact with titanium or zirconia was used as a control group. Using a cell strainer splenocytes were separated from the connecting tissue. Then, erythrocytes were removed by lysis and the remaining leukocytes were used for in vitro experiments. Using spleen cells will give a test for the immunogenicity of the material for proving the tolerance against the foreign material. The in vitro stimulation tests were performed with 2 × 10^6^ rat spleen cells/ml in a 24-well plate (Corning, Amsterdam, Netherlands) and incubated at 37 °C and with 5% CO_2_ for 3 and 24 h. The stimulation levels of the rat spleen cells for IL-6 and TNFα were assessed for four different materials. These included phytohemagglutine (PHA, PAN-Biotech GmbH, Aidenbach, Germany), lipopolysaccharide (LPS, Sigma-Aldrich Chemie GmbH, Munich, Germany), titaniumoxide nanopowder (Titanium(IV)oxide nanopowder, < 100 nm particle size, Sigma-Aldrich Chemie GmbH, Munich, Germany. and zirconium oxide nanopowder (Zirconium(IV)oxide nanopowder, < 100 nm particle size, Sigma-Aldrich Chemie GmbH, Munich, Germany). The following sample quantities were used (PHA: 2,5 µg/ml, LPS: 250 ng/ml, Titanium Oxide: 100 mg/ml, Zirconium Oxide: 100 mg/ml). Stimulation levels were measured after 3 and 24 h with BD Rat ELISA Sets for IL-6 and TNF (BD Biosciences Pharmingen, San Diego, CA, USA).

### Statistical analysis

The sample size was calculated using the nQuery Advisor software (Statsols, Version 8, Cork, Ireland) with McNemar’s test on the equality of paired samples. Using a p = 0.05 significant level, an odds ratio of 0.15^30^, and a power of 80%^36^, a group comparison of the target main study parameter produced a sample size of N = 18 rats per group, including two dropouts.

Analyses were performed using Prism 8 software for Mac OS X (GraphPad, La Jolla, CA, USA) running on Apple OS X. The analysis values were tested for normal distribution using the Kolmogorov–Smirnov normality test. Data for immunological parameters over time was analyzed using a mixed-effects model with the Geisser-Greenhouse correction. Furthermore, the Tukey’s multiple comparisons test and the intra-group comparison was assessed with the Kruskal–Wallis test and Dunn’s multiple comparisons test. Values of hypersensitivity reactions were evaluated using a two-way analysis of variance (ANOVA) and a Sidak’s multiple comparison test. P-values < 0.05 were considered statistically significant.

### Approval for animal experiments

For experiments involving live vertebrates and/or higher invertebrates, your Methods section must include a statement that:

- Identifies the institutional and/or licensing committee that approved the experiments, including any relevant details.
- Confirms that all experiments were performed in accordance with relevant named guidelines and regulations.
- We confirm that all experiments were performed in accordance with relevant named guidelines and regulations and the study complied with the ARRIVE guidelines.

## Results

### In vivo immunological and clinical examination

Of 54 rats, 52 were included in the evaluation. Two animals from the group zoledronic acid were lost, one in the course of the drug administration in the rat restrainer and the other in the anesthesia operation, probably to respiratory arrest.

**Figure 3:**
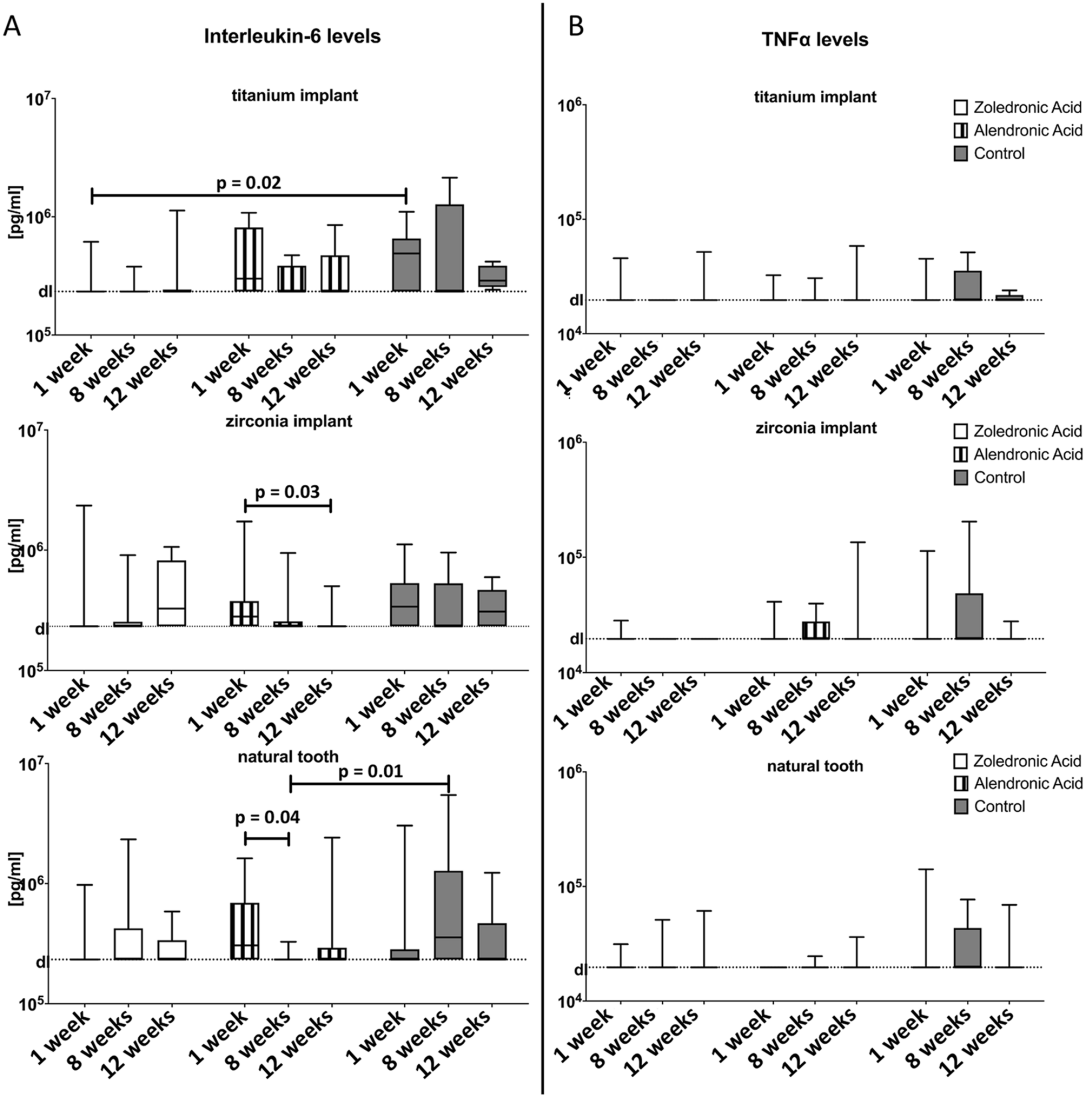
The evaluation for interleukin-6 and TNFα levels of both implant materials and the natural tooth were presented. The collection took place at three sessions: 1 week, 8 weeks, and 12 weeks after surgery. The detection limit was assessed for IL-6 at 233 ng/ml and for TNFα at 19.6 ng/ml after correction with dilution and recovery factor.

Inter-group analysis around titanium implants regarding IL-6 demonstrated significantly higher values at session 1 (one week after surgery) in the control group when compared to the zoledronic acid group (Fig. 3A, p = 0.02). However, the natural tooth in the control group at session 2 (8 weeks after surgery) presented higher values when compared to the alendronic acid group (p = 0.01). The lowest standard parameter (detection limit) was assessed for IL-6 at 233 (ng/ml) and for TNFα at 19.6 (ng/ml). Within each group over time between sessions 1 and 3 (1 and 12 weeks after surgery) around zirconia implants (p = 0.03) and between sessions 1 and 2 (one and 8 weeks after surgery) around teeth (p = 0.04) with alendronic acid drug administration, a significant reduction of IL-6 was observed (Fig. 3A). Furthermore, comparisons of subgroups over time showed a significant difference at session 2 (8 weeks after surgery) between the titanium implants of the zoledronic acid group and the teeth of the control group (p = 0.03).

Inter- and intra-group analysis for TNFα values presented no significant differences within each subgroup, such as with or without drug administration, for both implant materials and the natural teeth (Fig. 3B, p > 0.05).

**Figure 4:**
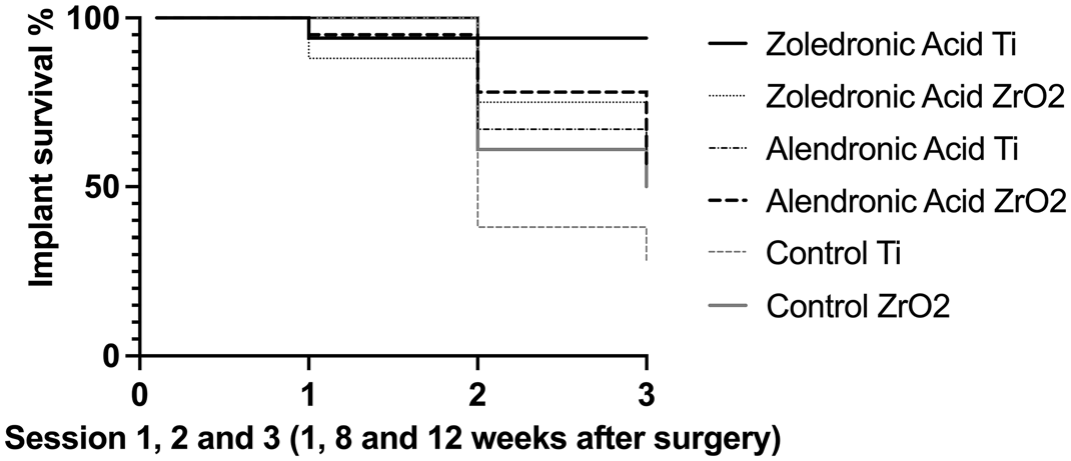
Presentation of the survival chart regarding all groups and implant materials (Ti = titanium, ZrO2 = zirconia).

After 3 months of follow-up, for titanium implants in the zoledronic acid group, a survival rate of 93.00% with a success rate of 60.00% was observed. For the alendronic acid group, it was a survival rate of 55.60% with a success rate of 70.00%, and in the control group without drug administration, it was a survival rate of 37.00% with a success rate of 100% (Table 1, Fig. 4). On the other hand, zirconia implants demonstrated in the zoledronic acid group a survival rate of 75.00% with a success rate of 41.60%; in the alendronic acid group, a survival rate of 55.60% with a success rate of 54.00%; and in the control group without drug administration, a survival rate of 61.20% with a success rate of 72.72%.

**Table 1:** Survival and success rates (%) were calculated for titanium and zirconia implants.

	Group	Zoledronic Acid	Alendronic Acid	Control
Survival rate %	**Session I (one week after surgery)**			
	Zirconia	87.50	95.40	100.00
	Titanium	93.70	100.00	100.00
	Total	90.60	97.70	100.00
	**Session II (8 weeks after surgery)**			
	Zirconia	75.00	77.80	61.20
	Titanium	93.70	66.70	37.80
	Total	84.35	72.25	38.90
	**Session III (12 weeks after surgery)**			
	Zirconia	75.00	55.60	50.00
	Titanium	93.70	55.60	27.80
	Total	84.35	55.60	49.50
Success rate %	**Session III (12 weeks after surgery)**			
	Zirconia	41.60	54.00	72.72
	Titanium	60.00	70.00	100.00
	Total	50.80	62.00	86.36

Regarding the PPD around teeth in groups with and without drug application, mean values of approximately 1 mm were comparable (Table 2). However, around both implant materials, PPD values were measured at approximately 2 mm in both test groups. Mean SBI value over all subgroups and implant materials and natural teeth demonstrated low inflammation below 0.5 (score between 0 = no bleeding and 1 = isolated bleeding).

**Table 2:** Clinical parameters at session III (12 weeks after surgery) included the pocket probing depth (PPD) and the sulcus bleeding index (SBI) around each implant or tooth (PDD = pocket probing depth, SBI = sulcus bleeding index, SD = standard deviation).

Session III	PDD				SBI		
	Zoledronic Acid	Alendronic Acid	Control		Zoledronic Acid	Alendronic Acid	Control
**Zirconia**							
Mean	2.00	2.00	1.18	Mean	0.33	0.09	0.27
SD	0.95	0.89	0.40	SD	0.49	0.30	0.47
**Titanium**							
Mean	2.07	1.90	1.20	Mean	0.13	0.00	0.00
SD	0.80	0.88	0.45	SD	0.35	0.00	0.00
**Tooth**							
Mean	1.25	1.33	1.06	Mean	0.00	0.00	0.06
SD	0.44	0.48	0.23	SD	0.00	0.00	0.23

### In vitro immunological examination

**Figure 5:**
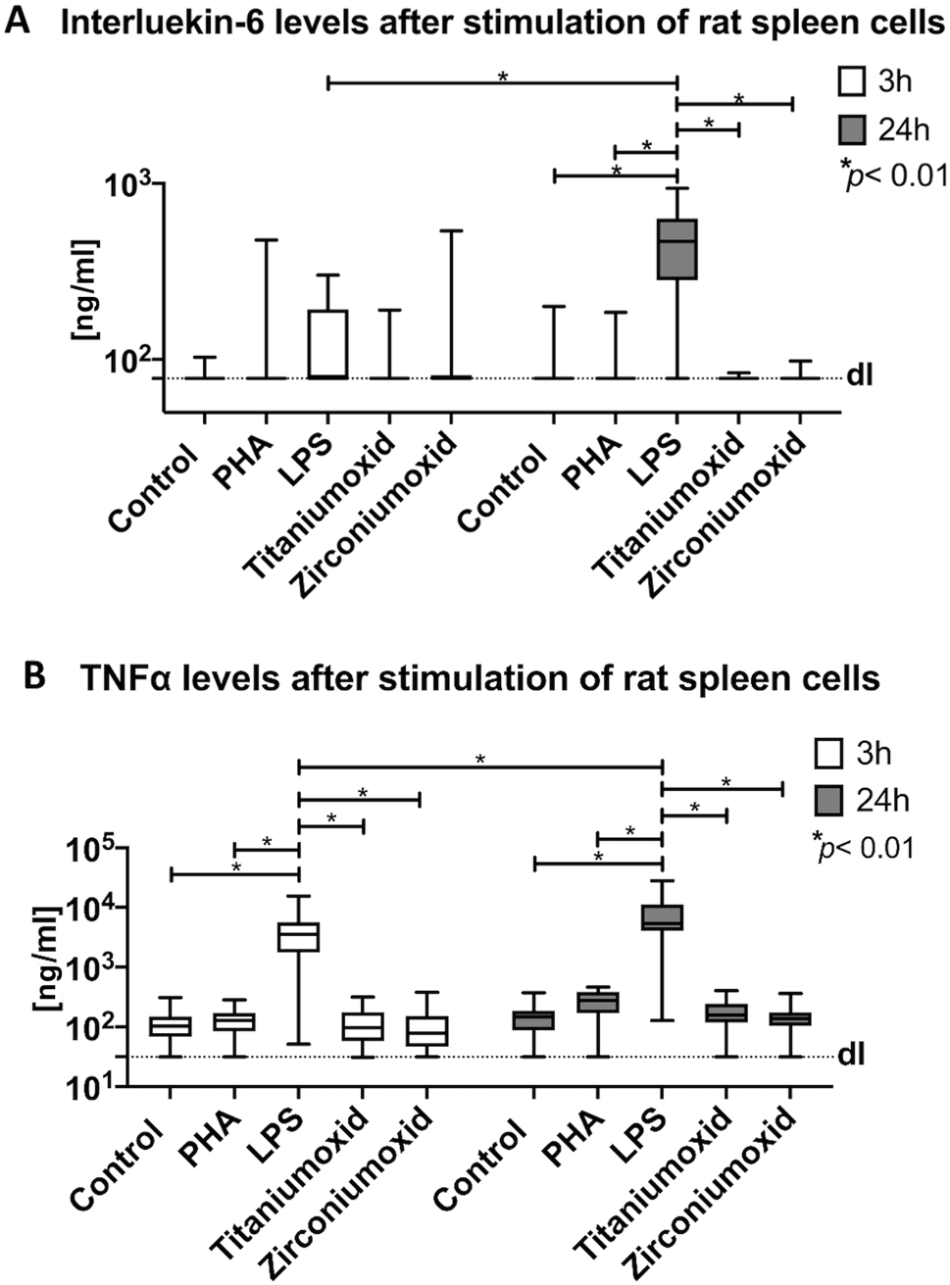
The stimulation levels of the rat spleen cells for interleukin-6 and TNFα were shown for phytohemagglutinine (PHA), lipopolysaccharide (LPS), titaniumoxid-nanopowder and zirconiumoxide nanopowder in relation to a control group. Stimulation levels were assessed after 3 and 24 h. The lowest standard parameter (detection limit) was assessed for IL-6 at 0.078 ng/ml and for TNFα at 0.031 ng/ml.

Between the IL-6 measurements at 3 and 24 h time points after stimulation, only LPS stimulation presented a significant IL-6 value increase. When compared to all other groups (control, PHA, titaniumoxide powder, and zirconiumoxide powder), only intra-material analysis 24 h after LPS stimulation of the rat spleen cells revealed a significantly higher IL-6 parameter (Fig. 5A, p < 0.01). TNFα values were comparable to those of IL-6, as LPS stimulation significantly increased TNFα levels between 3 and 24 h (p < 0.01). Additionally, intra-material analysis at 3 h and again at 24 h showed a significantly higher TNFα production after LPS stimulation (Fig. 5B, p < 0.01). The lowest standard parameter (detection limit) was assessed for IL-6 at 0.078 ng/ml and for TNFα at 0.031 ng/ml.

## Discussion

The aim of this rat study was to evaluate the host-inflammatory response of peri-implant interfaces around titanium and zirconia implants in a high-risk group with systematic antiresorptive bisphosphonate drug administrations. Evidence showed that IL-6 polymorphisms were involved in soft tissue inflammation^37,38^. Furthermore, the significantly higher levels of TNFα in peri-implantitis patients indicated that TNFα also played a key role in peri-implantitis and that TNFα was a proinflammatory cytokine that promoted the resorption of the bone and mediates the infection's inflammatory response^27^. In this experiment, we sought to determine how the chosen drug administrations and the different materials affected these inflammatory parameters. Our results indicated that IL-6 demonstrated significantly higher values in the control groups without drug administration around titanium implants and the natural tooth. A possible explanation for the increased IL-6 release in the healthy control group could possibly be a better and faster reacting bone metabolism and thus more pronounced inflammatory reaction to a high-risk implant procedure. However, analysis for TNFα values presented no significant differences within each subgroup. Furthermore, in an in vivo study in humans, titanium and zirconia implants performed better than their natural counterparts in terms of IL-6 and TNFα^25^. By contrast, another mucositis study suggested that the implant group had a significantly higher expression of IL-6 than the healthy tooth group^39^.

With regard to TNFα, published data are quite controversial. On the one hand, the key role of TNFα in peri-implantitis was indicated by significantly higher levels of TNFα in patients with peri-implantitis compared to those in the control group^27^. Other analyses found that the risk of dental peri-implant disease was not significantly associated with TNFα-polymorphism^40,41^.

In terms of survival, the control group had the lowest and the zoledronic group the highest survival rates. However, the success rate was reversed, with the control group having the highest success rate and the zoledronic group the lowest. Data from seven studies enabled comparing the survival rate of titanium implants in a meta-analysis^42^. The survival rates up to 12 months after loading were very high (> 98%). Similarly, for the material titanium, a current meta-analysis estimated the 1- and 2-year survival rates for dental zirconia implants inserted in humans to be 98.3% and 97.2%, respectively^43^. These findings agree with the study of Adanez et al., which suggested that the mean survival rate of zirconia implants was 95%^44^. Intravenous administration, as applied in this study, is associated with a higher risk of peri-implant inflammation and might be the reason for poor success rates^45^. A possible cause for the reduced success could be the drug reduced bone remodeling^3^. It is evident that bisphosphonates inhibit osteoclasts and thus negatively affect not only the blood supply to the peri-implant bone but also the healing capacity^3,7,8^. In the case of inflammation, therefore, a reduced blood supply and immune defense would have to be assumed. Further studies should include a follow-up longer than 3 months, as it should be assumed that the bisphosphonate groups may demonstrate higher implant loss rates due to the adverse success rates.

Besides the in vivo immunological findings of this study, the stimulation effect of titanium and zirconia nanoparticles on host inflammatory parameters was also assessed in an in vitro set-up. Patients may be exposed internally to nanoparticles by wear mechanisms associated with dental implants^46^. When macrophages phagocytose these nanoparticles, they release many pro-inflammatory and pro-osteoclastogenic cytokines, such as TNFα and IL-6, to promote osteoclastogenesis^46^. Wear is not expected directly after insertion but low product quality may be a reason for particle release after implant insertion^47,48^. If released particles affect implant performance is not clear yet. Zhang et al. demonstrated that zirconia nanoparticles were bioactive to cells, and their results indicated that such nanoparticles might be more irritating to macrophages than titanium microparticles^49^. In addition to the effects of zirconia nanoparticles, effects of titanium particles have also been described^50^. Although, like zirconia, the block biocompatibility properties of titanium are excellent^51^, the adverse effects of titanium particles on osteoblast function have been noted^52^. Our in vitro stimulation tests suggested the biocompatibility of both titanium and zirconia nanoparticles with rat cells. Furthermore, He et al. revealed that titanium and zirconia dental implants demonstrated titanium and zirconia concentrations in the bone tissue of porcine jaws^53^. The titanium content released by titanium implants was twice as high as the zirconia content released by zirconia implants^53^. In addition, zirconia nanoparticles presented lower cytotoxicity and DNA damage compared to the results reported for titanium nanoparticles in human cells. The current data situation regarding the exact chemical composition of nanoparticles in peri-implant tissue (either zirconia or titanium particles) is currently unclear. Based on conventional elemental analysis, it is currently not possible to say in which chemical form the nanoparticles are actually present in the tissue (oxidized, ionized, elemental). This should be considered when interpreting the data”.

A limitation of this study was that the split-mouth design was lost in several animals, as they lost one or more implants. Additionally, we recognize that IL-6 and TNFα metabolism can vary between our rat model and humans. For future studies immunohistochemical results for the tissue-implant-interface are a very interesting study design approach, as local presence of IL-6 and TNF-a within the tissue-implant interface may differ between paper point collection levels. When interpreting the results, it should be noted that the soft- and not the hard tissue was analyzed.

## Conclusions

Regarding in vivo and in vitro IL-6 and TNFα production, no implant material was superior to the other. No significant in vitro stimulation of rat spleen cells was detected with respect to titanium oxide and zirconium oxide nanoparticles. Systemic bisphosphonate delivery led to decreased implant success after up to 3 months of follow-up. However, in terms of implant survival, the control group had the lowest rate and the zoledronic group the highest. According to our data no better prognosis could be achieved with antiresorptive therapy using alternative implant materials than with standard titanium implants. Therefore, the indication cannot be shifted more clearly in favor of implant placement.

## Supplementary Information

Supplementary Information.
