# Supplementary material for: Host inflammatory response and clinical parameters around implants in a rat model using systemic alendronate and zoledronate acid drug administrations
Source: Sci Rep. 2022 Mar 15;12:4431. doi: 10.1038/s41598-022-08308-8 (PMC8924183; doi:10.1038/s41598-022-08308-8)
Supplement: Supplementary file 1 — Supplementary Information. [file 41598_2022_8308_MOESM1_ESM.docx]

| **Supplementary Table 1**  Descriptive statistics of in vivo and in vitro parameters | | | | | | | | | | | | | | | | |
| --- | --- | --- | --- | --- | --- | --- | --- | --- | --- | --- | --- | --- | --- | --- | --- | --- |
|  |  |  | in vivo parameters | | | | | | | | | | |  |  | |
|  |  |  | titanium | | |  | zirconia | | |  | natural tooth | | |  |  |  |
|  | Il-6 |  | Zoledronic Acid | Alendronic Acid | Control |  | Zoledronic Acid | Alendronic Acid | Control |  | Zoledronic Acid | Alendronic Acid | Control |  |  |  |
| session I |  | Total number of measurments | 92 | 84 | 54 |  | 81 | 89 | 79 |  | 96 | 108 | 108 |  |  |  |
|  |  | Minimum | 2,33E+05 | 2,33E+05 | 2,33E+05 |  | 2,33E+05 | 2,33E+05 | 2,33E+05 |  | 2,33E+05 | 2,33E+05 | 2,33E+05 |  |  |  |
|  |  | Maximum | 6,13E+05 | 1,08E+06 | 1,10E+06 |  | 2,35E+06 | 1,73E+06 | 1,12E+06 |  | 9,75E+05 | 1,62E+06 | 3,04E+06 |  |  |  |
|  |  | Range | 3,80E+05 | 8,47E+05 | 8,67E+05 |  | 2,12E+06 | 1,50E+06 | 8,82E+05 |  | 7,42E+05 | 1,39E+06 | 2,81E+06 |  |  |  |
|  |  | Mean | 2,64E+05 | 4,78E+05 | 5,00E+05 |  | 4,01E+05 | 3,85E+05 | 4,20E+05 |  | 3,23E+05 | 5,07E+05 | 4,37E+05 |  |  |  |
|  |  | Std. Deviation | 9,74E+04 | 3,04E+05 | 2,59E+05 |  | 5,49E+05 | 3,48E+05 | 2,32E+05 |  | 2,12E+05 | 4,26E+05 | 6,59E+05 |  |  |  |
|  |  | Std. Error of Mean | 2,44E+04 | 7,15E+04 | 6,29E+04 |  | 1,42E+05 | 8,21E+04 | 5,47E+04 |  | 5,31E+04 | 1,00E+05 | 1,55E+05 |  |  |  |
|  |  |  |  |  |  |  |  |  |  |  |  |  |  |  |  |  |
|  | TNFa | Minimum | 1,97E+04 | 1,97E+04 | 1,97E+04 |  | 1,97E+04 | 1,97E+04 | 1,97E+04 |  | 1,97E+04 | 1,97E+04 | 1,97E+04 |  |  |  |
|  |  | Maximum | 4,56E+04 | 3,24E+04 | 4,52E+04 |  | 2,83E+04 | 4,12E+04 | 1,13E+05 |  | 3,13E+04 | 1,97E+04 | 1,41E+05 |  |  |  |
|  |  | Range | 2,60E+04 | 1,28E+04 | 2,55E+04 |  | 8,65E+03 | 2,15E+04 | 9,37E+04 |  | 1,17E+04 | 0,00E+00 | 1,21E+05 |  |  |  |
|  |  | Mean | 2,13E+04 | 2,04E+04 | 2,26E+04 |  | 2,06E+04 | 2,16E+04 | 3,02E+04 |  | 2,04E+04 | 1,97E+04 | 2,92E+04 |  |  |  |
|  |  | Std. Deviation | 6,49E+03 | 3,01E+03 | 7,15E+03 |  | 2,47E+03 | 5,64E+03 | 3,02E+04 |  | 2,91E+03 | 0,00E+00 | 3,03E+04 |  |  |  |
|  |  | Std. Error of Mean | 1,62E+03 | 7,09E+02 | 1,73E+03 |  | 6,39E+02 | 1,33E+03 | 7,12E+03 |  | 7,28E+02 | 0,00E+00 | 7,15E+03 |  |  |  |
|  |  |  |  |  |  |  |  |  |  |  |  |  |  |  |  |  |
| session II | Il-6 | Minimum | 2,33E+05 | 2,33E+05 | 2,33E+05 |  | 2,33E+05 | 2,33E+05 | 2,33E+05 |  | 2,33E+05 | 2,33E+05 | 2,33E+05 |  |  |  |
|  |  | Maximum | 3,78E+05 | 4,73E+05 | 2,14E+06 |  | 9,08E+05 | 9,46E+05 | 9,56E+05 |  | 2,34E+06 | 3,27E+05 | 5,47E+06 |  |  |  |
|  |  | Range | 1,45E+05 | 2,40E+05 | 1,91E+06 |  | 6,74E+05 | 7,13E+05 | 7,23E+05 |  | 2,10E+06 | 9,41E+04 | 5,24E+06 |  |  |  |
|  |  | Mean | 2,49E+05 | 2,94E+05 | 6,49E+05 |  | 3,30E+05 | 2,93E+05 | 3,92E+05 |  | 4,67E+05 | 2,43E+05 | 9,94E+05 |  |  |  |
|  |  | Std. Deviation | 4,25E+04 | 9,88E+04 | 8,37E+05 |  | 2,25E+05 | 1,83E+05 | 2,60E+05 |  | 5,51E+05 | 2,92E+04 | 1,32E+06 |  |  |  |
|  |  | Std. Error of Mean | 1,10E+04 | 2,74E+04 | 3,74E+05 |  | 6,48E+04 | 4,72E+04 | 7,84E+04 |  | 1,38E+05 | 6,88E+03 | 3,11E+05 |  |  |  |
|  |  |  |  |  |  |  |  |  |  |  |  |  |  |  |  |  |
|  | TNFa | Minimum | 1,97E+04 | 1,97E+04 | 1,97E+04 |  | 1,97E+04 | 1,97E+04 | 1,97E+04 |  | 1,97E+04 | 1,97E+04 | 1,97E+04 |  |  |  |
|  |  | Maximum | 1,97E+04 | 3,06E+04 | 5,14E+04 |  | 1,97E+04 | 3,98E+04 | 2,04E+05 |  | 5,11E+04 | 2,46E+04 | 7,68E+04 |  |  |  |
|  |  | Range | 0,00E+00 | 1,09E+04 | 3,17E+04 |  | 0,00E+00 | 2,02E+04 | 1,85E+05 |  | 3,14E+04 | 4,91E+03 | 5,72E+04 |  |  |  |
|  |  | Mean | 1,97E+04 | 2,07E+04 | 2,60E+04 |  | 1,97E+04 | 2,41E+04 | 4,63E+04 |  | 2,17E+04 | 1,99E+04 | 3,07E+04 |  |  |  |
|  |  | Std. Deviation | 0,00E+00 | 3,04E+03 | 1,42E+04 |  | 0,00E+00 | 7,66E+03 | 5,49E+04 |  | 7,84E+03 | 1,16E+03 | 1,96E+04 |  | in vitro stimulation tests | |
|  |  | Std. Error of Mean | 0,00E+00 | 8,13E+02 | 6,35E+03 |  | 0,00E+00 | 2,13E+03 | 1,65E+04 |  | 1,96E+03 | 2,73E+02 | 4,62E+03 |  | 3h | 24h |
|  |  |  |  |  |  |  |  |  |  |  |  |  |  | number | 90 | 90 |
| session III | Il-6 | Minimum | 2,33E+05 | 2,33E+05 | 2,41E+05 |  | 2,33E+05 | 2,33E+05 | 2,33E+05 |  | 2,33E+05 | 2,33E+05 | 2,33E+05 |  | 7,80E+01 | 7,80E+01 |
|  |  | Maximum | 1,13E+06 | 8,52E+05 | 4,17E+05 |  | 1,06E+06 | 5,04E+05 | 5,98E+05 |  | 5,86E+05 | 2,42E+06 | 1,23E+06 |  | 5,38E+02 | 9,39E+02 |
|  |  | Range | 8,94E+05 | 6,19E+05 | 1,76E+05 |  | 8,30E+05 | 2,71E+05 | 3,65E+05 |  | 3,52E+05 | 2,18E+06 | 9,98E+05 |  | 4,60E+02 | 8,61E+02 |
|  |  | Mean | 3,13E+05 | 3,43E+05 | 3,13E+05 |  | 4,84E+05 | 2,66E+05 | 3,54E+05 |  | 2,86E+05 | 4,32E+05 | 3,88E+05 |  | 1,04E+02 | 1,64E+02 |
|  |  | Std. Deviation | 2,35E+05 | 2,05E+05 | 7,11E+04 |  | 3,11E+05 | 8,35E+04 | 1,38E+05 |  | 9,67E+04 | 5,63E+05 | 2,58E+05 |  | 7,53E+01 | 1,98E+02 |
|  |  | Std. Error of Mean | 6,06E+04 | 6,47E+04 | 3,18E+04 |  | 8,99E+04 | 2,32E+04 | 4,35E+04 |  | 2,42E+04 | 1,37E+05 | 6,09E+04 |  | 7,94E+00 | 2,08E+01 |
|  |  |  |  |  |  |  |  |  |  |  |  |  |  |  |  |  |
|  | TNFa | Minimum | 1,97E+04 | 1,97E+04 | 1,97E+04 |  | 1,97E+04 | 1,97E+04 | 1,97E+04 |  | 1,97E+04 | 1,97E+04 | 1,97E+04 |  | 3,13E+01 | 3,13E+01 |
|  |  | Maximum | 5,18E+04 | 5,84E+04 | 2,39E+04 |  | 1,97E+04 | 1,35E+05 | 2,79E+04 |  | 6,11E+04 | 3,62E+04 | 6,92E+04 |  | 1,55E+04 | 2,81E+04 |
|  |  | Range | 3,21E+04 | 3,87E+04 | 4,26E+03 |  | 0,00E+00 | 1,15E+05 | 8,21E+03 |  | 4,15E+04 | 1,65E+04 | 4,95E+04 |  | 1,55E+04 | 2,81E+04 |
|  |  | Mean | 2,21E+04 | 2,32E+04 | 2,05E+04 |  | 1,97E+04 | 3,26E+04 | 2,04E+04 |  | 2,43E+04 | 2,06E+04 | 2,24E+04 |  | 9,19E+02 | 1,84E+03 |
|  |  | Std. Deviation | 8,25E+03 | 1,17E+04 | 1,91E+03 |  | 0,00E+00 | 3,42E+04 | 2,48E+03 |  | 1,13E+04 | 4,01E+03 | 1,17E+04 |  | 2,25E+03 | 4,80E+03 |
|  |  | Std. Error of Mean | 2,13E+03 | 3,52E+03 | 8,52E+02 |  | 0,00E+00 | 9,88E+03 | 7,47E+02 |  | 2,82E+03 | 9,72E+02 | 2,75E+03 |  | 2,37E+02 | 5,06E+02 |
